# Aqueous Extracts from Tunisian *Diplotaxis*: Phenol Content, Antioxidant and Anti-Acetylcholinesterase Activities, and Impact of Exposure to Simulated Gastrointestinal Fluids

**DOI:** 10.3390/antiox5020012

**Published:** 2016-04-02

**Authors:** Nada Bahloul, Sana Bellili, Smail Aazza, Ameur Chérif, Maria Leonor Faleiro, Maria Dulce Antunes, Maria Graça Miguel, Wissem Mnif

**Affiliations:** 1 LR11-ES31 Laboratory of Biotechnology and Valorisation of Bio-GeoRessources (BVBGR), Higher Institute of Biotechnology of Sidi Thabet (ISBST), University of Manouba, Biotechpole Sidi Thabet, Ariana 2020, Tunisia; nada.bahloul@gmail.com (N.B.); bel-sana@hotmail.fr (S.B.); cherif.ameur@gmail.com (A.C.); w_mnif@yahoo.fr (W.M.); 2 Faculty of Sciences of Bizerte, University of Carthage, Jarzouna-Bizerte 7021, Tunisia; 3 Laboratory of Physiology-Pharmacology-Environmental Health, Faculty of Sciences Dhar El Mehraz, BP 1796 Atlas, University Sidi Mohamed Ben Abdallah, Fez 30 000, Morocco; aazzasmail@ymail.com; 4 Faculdade de Ciências e Tecnologia, Center for Biomedical Research, Universidade do Algarve, Edf. 8, Campus de Gambelas, Faro 8005-139, Portugal; mfaleiro@ualg.pt; 5 Faculdade de Ciências e Tecnologia, Universidade do Algarve, MeditBio, Edif. 8, Campus de Gambelas, Faro 8005-139, Portugal; mantunes@ualg.pt; 6 Faculty of Sciences and Arts in Balgarn PO BOX 60 Balgarn, Bisha University, Sabt Al Alaya 61985, Saudi Arabia

**Keywords:** *Diplotaxis harra*, *Diplotaxis simplex*, biological activities, digestion

## Abstract

Antioxidants have been considered essential for preventing cell damage by scavenging deleterious free radicals. The consumption of antioxidant-rich plants is associated with a reduced risk of some chronic diseases. This study evaluates the antioxidant and acetylcholinesterase inhibition activities of aqueous extracts obtained from different parts of *Diplotaxis simplex* and *Diplotaxis harra* from Tunisia. The study also aimed to investigate the action of simulated gastrointestinal juice on antioxidant activities of both extracts. The total phenolic, flavone and flavonol, and flavanone and dihydroflavonol contents were determined by Folin–Ciocalteau, aluminum chloride and 2,4-dinitrophenylhydrazine colorimetric methods, respectively. The metal ion chelating activity, acetylcholinesterase inhibition capacity, and free radical scavenging potential of the extracts towards ABTS (2,2′-azinobis-(3-ethylbenzothiazoline-6-sulfonic acid), DPPH (2,2-diphenyl-1-picrylhydrazyl), hydroxyl, superoxide and nitric oxide were also evaluated. The action of simulated gastro-intestinal fluids on the flavone and flavonol content and total antioxidant activity of the flower extracts was surveyed. Extracts from the seeds and flowers of *D. simplex* and *D. harra* displayed the highest amounts of phenols (2691.7 and 2694.5 mg Caffeic Acid Equivalent (CAE)/100 mg; 3433.4 and 2647.2 mg CAE/100 mg, respectively) and flavonols/flavones (2144.4 and 2061.1 mg Rutin Equivalent (RE)/100 g; 1922.6 and 1461.1 mg RE/100 g, respectively). The flower and seed extracts exhibited the highest rates of antioxidant and acetylcholinesterase inhibition activities. A decrease in the flavonoid content and antioxidant activity was observed after extract exposure to simulated saliva. Antioxidant and acetylcholinesterase inhibition activities were noted to depend on plant species and plant parts. *In vitro* gastrointestinal digestion is useful in assessing the bio-accessibility of compounds with biological activities from food. The simulated gastrointestinal fluids influenced the flavonoid concentration and antioxidant activity.

## 1. Introduction

*Diplotaxis* DC (Brassicaceae, tribe Brassiceae) is a large genus of flowering plants in the *Brassicaceae* family. It comprises more than 30 species, native to Europe, the Mediterranean basin, SW Asia and Macaronesia [1,2]. Glucosinolates, flavonoids and proteins have been reported in the taxa [1,3,4,5,6,7,8]. *Diplotaxis* species present a high level of heterogeneity in morphology and geographical distribution. The geographical distribution of *Diplotaxis harra* (Forssk.) Boiss. is North Africa and Middle East, and for *D. simplex* (Viv.) Spr. (syn. *Diplotaxis muralis* subsp. *simplex* (Viv.) Jafri) is Algeria, Tunisia, Libya, and Egypt [9].

*Diplotaxis* species have been widely used for various purposes, ranging from food to cosmetic and medicinal uses. Some species of *Diplotaxis* (*D. tenuifolia* (L.) DC, *D. muralis* (L.) DC, *D. harra*) are widely consumed as rocket salad, while others are considered as arable weeds (*D. erucoides* (L.) DC) or sources of healthy phytochemicals [2,10,11]. Falleh *et al.* [12] have reported on the rich phenol content and attractive antioxidant and antibacterial activities of methanolic extracts obtained from the stems, leaves and flowers of *D. simplex* and *D. harra*. The phenolic content as well as the antioxidant activity found by the authors depended not only on the species and their organs (flowers, leaves, stems) but also on the method used. For example, the capacity for reducing Mo(VI) to Mo(V) was better in flower extracts of *D. harra*, whereas the capacity for reducing Fe(III) to Fe(II) was better in methanolic leaf extracts of *D. simplex*. The antioxidant activity may have an important role in the prevention of diseases caused by oxidative stress such as neurodegenerative processes, namely Alzheimer’s disease. Besides the oxidative stress, other pathogenic factors are involved in this disease such as the reduced acetylcholine levels [13]. The inhibition of acetylcholinesterase, which hydrolyses acetylcholine to choline and acetate, gives rise to an increased acetylcholine concentrations in the synaptic cleft and consequently an enhanced cholinergic transmission [13].

Not all food bioactive phytochemicals are absorbed by the gastrointestinal tract. In this way, only a small proportion of these compounds is available to exert their biological functions. Bio-accessibility corresponds to the fraction of bioactive compounds that is released from the food matrix after digestion and solubilisation into the gut lumen for uptake in the intestinal mucosa, whereas a bio-availability is defined as the fraction of nutrient secreted into circulation that is available for tissue uptake and metabolism [14].

In general, *in vitro* gastrointestinal digestion is useful in assessing the bio-accessibility of compounds with biological activity from food [15]. There are some studies assessing the *in vitro* bio-accessibility of bioactive compounds in simple beverages [15].

Considering the promising opportunities that *Diplotaxis*-derived extracts might offer for the continuous search for plant-derived bioactive compounds to substitute conventional chemical compounds, the present study was undertaken to investigate the phenol content, metal ion chelating activities, acetylcholinesterase inhibition capacity, and free radical scavenging potential of aqueous extracts from the stems, leaves, flowers, roots, siliques, and seeds of Tunisian *D. simplex* and *D. harra* species. The present study explores the availability of potential phytochemicals present in the samples and their ability to exert antioxidant and antiacetylcholinesterase activities. The study also examined, for the first time, the action of simulated gastro-intestinal fluids on the flavone and flavonol content and total antioxidant activities of the aqueous extracts.

## 2. Materials and Methods 

### 2.1. Plant Material

*D. simplex* and *D. harra* were collected in March, 2014 from the area of Sfax (South Tunisia, arid climate). The plant specimens were identified by Prof. Mohamed Chaieb, botanist in the Faculty of Sciences of Sfax—Tunisia. All samples were shade dried for 15 days at room temperature with ventilation. The material was cut into small pieces and powdered (roots, stems, leaves, flowers and siliques) in an electric blender. The powders were collected and stored at room temperature away from moisture before analysis.

### 2.2. Plant Extraction

The extracts were prepared using the following procedures. A decoction was prepared by placing 5 g of dried plant material for 30 min in 100 mL of distilled water. The samples were then centrifuged for 5 min at 2000 g and 20 °C, and the supernatant was removed and kept at −20 °C until further assays.

### 2.3. Determination of Total Phenols (Folin–Ciocalteau)

The total phenol content in the extracts was determined, using the Folin–Ciocalteau reagent (Panreac Química, Montcada i Reixac, Barcelona, Spain) as described by Slinkard and Singleton [16] and caffeic acid (Fluka. Biochemika, Sigma-aldrich, Steinheim, Germany) used as a standard. In brief, the sample (0.5 mL) and 2 mL of sodium carbonate (Riedel de Haen, Seelze, Germany) (75 g/L) were added to 2.5 mL of 10% (*v/v*) Folin–Ciocalteau reagent. After 30 min of reaction at room temperature, absorbance was measured at 765 nm. Tests were carried out in triplicate and presented as mg Caffeic Acid Equivalent (CAE)/100 g extract.

### 2.4. Flavone and Flavonol Content 

Flavonol and flavone contents were quantified as previously described [17]. Briefly, 0.5 mL of 2% AlCl_3_-ethanol (Panreac Química, Montcada i Reixac, Barcelona, Spain) solution were added to 0.5 mL of the sample or standard. After 1 h of reaction at room temperature, absorbance was measured at 420 nm. Rutin (Fluka Biochemika, Sigma-Aldrich) was used as a standard for the construction of calibration curves. The results are presented as mg Rutin Equivalent/100 extract (mg rutin RE/100 g).

### 2.5. Flavanone and Dihydroflavonol Content

The total flavanone and dihydroflavonol contents were measured as previously described in the literature [18]. In brief, an aliquot (1 mL) of sample or standard and 2 mL of DNP (2,4-dinitrophenylhydrazine) (Fluka Biochemika, Sigma-Aldrich) solution (1 g DNP in 2 mL 96% sulphuric acid (Fluka Biochemika, Sigma-Aldrich,), diluted to 100 mL with methanol) were heated at 50 °C for 50 min. The mixture was diluted to 10 mL with 10% KOH (Fluka Biochemika, Sigma-aldrich) in methanol (*w/v*) (Merck, Darmstadt, Germany) after cooling at room temperature. A sample mixture (1 mL of the resulting solution) was added to 10 mL methanol and diluted to 50 mL with methanol. Absorbance was measured at 486 nm.

### 2.6. ABTS^•^^+^ Free Radical-Scavenging Activity

ABTS^•^^+^ (Fluka Biochemika, Sigma-Aldrich) radical scavenging activity was determined as previously described [19]. The sample concentration providing IC_50_ was obtained by plotting the inhibition percentage against extract concentrations. Tests were carried out in triplicate.

### 2.7. DPPH Free Radical-Scavenging Activity

A methanolic stock solution (50 µL) of each sample at different concentrations was placed in a cuvette, and 2 mL of a 60 µM methanolic solution of DPPH (2,2-diphenyl-1-picrylhydrazyl) (Riedel-de Haën, Sigma-Aldrich, Seelze, Germany) was added [20]. Absorbance was measured at 517 nm after 30 min of reaction at room temperature. IC_50_ values were determined as reported above. All tests were carried out in triplicate.

### 2.8. Chelating Metal Ions

The metal chelating activity of the sample extracts on ferrous ions was evaluated according to the method described by Wang *et al.* [21]. Tests were carried out in triplicate. The extract concentration providing IC_50_ was obtained by plotting the inhibition percentage against sample concentrations. 

### 2.9. Hydroxyl Radical Scavenging Activity

The OH-scavenging activity assay was performed according to a slightly modified version of the method described by Chung *et al.* [22]. In brief, a reaction mixture containing the sample extract, 10 mM FeSO_4_·7H_2_O (Fluka Chemicals, Buchs, Switzerland), 10 mM EDTA (Acros organics, Morris Plains, NJ, USA), 10 mM 2-deoxyribose, and 0.1 M phosphate buffer (Fisher Scientific, Fair Lawn, NJ, USA) was prepared in a test tube at total volume of 1.8 mL. After that, 200 µL of H_2_O_2_ (Panreac Quimica, Montcada i Reixac, Barcelona, Spain) were added to the test tube, and the mixture was incubated at 37 °C for 4 h. Subsequently, 1 mL trichloroacetic acid (2.8%) (Fluka Biochemika, Sigma-Aldrich, Steinheim, Germany) and 1 mL thiobarbituric acid (1%) (Fluka Biochemika, Sigma-Aldrich, Steinheim, Germany) were added, and the test tube was submitted to boiling for 10 min. After cooling, absorbance was measured at 520 nm using a Shimadzu 160-UV spectrophotometer (Tokyo, Japan). The OH-scavenging activity (%) was calculated using the following equation: Inhibition (%) ((*A*_0_ − *A*_1_)/*A*_0_)) × 100, where *A*_0_ is the absorbance of the control (without sample) and *A*_1_ is the absorbance in the presence of the sample. Tests were carried out in triplicate. The sample concentration providing IC_50_ was obtained by plotting the inhibition percentage against extract concentrations.

### 2.10. Superoxide Anion Scavenging Activity (Non-Enzymatic Method)

The superoxide anion scavenging activity of the samples and positive control were measured in accordance with the method described by Soares [23]. Superoxide anions were generated in a non-enzymatic phenazine methosulfatenicotinamide adenine dinucleotide (PMS-NADH) (Acros organics, Morris Plains, NJ, USA) system by NADH oxidation and assayed by the reduction of nitroblue tetrazolium (NBT) (Sigma Aldrich Chemie, Steinheim, Germany). Tests were carried out in triplicate. The sample concentration providing IC_50_ was obtained by plotting the inhibition percentage against extract concentrations.

### 2.11. Total Antioxidant Capacity (by Ammonium Molybdate Reduction Method)

Total antioxidant capacity was measured for different extract concentrations mixed with 1.5 mL of reagent solution (0.6 M sulphuric acid, 28 mM sodium phosphate and 4 mM ammonium molybdate) after 90 min of incubation at 95 °C. The samples were left to cool at room temperature, and absorbance was measured at 695 nm. Antioxidant activity was expressed as equivalents of ascorbic acid/100 g extract.

### 2.12. Nitric Oxide Scavenging Capacity

The nitric oxide (NO) scavenging activity of the samples was measured according to the method described by Ho *et al.* [24]. In brief, 50 µL of serially diluted sample were mixed with 50 µL of 10 mM sodium nitroprusside (Riedel-de Haën, Sigma-Aldrich, Seelze, Germany) in phosphate buffer saline (PBS) in a 96-well plate and incubated at room temperature for 90 min. Finally, an equal volume of Griess reagent (Promega Corporation, Madison, WI, USA) was added to each well, and absorbance was read at 546 nm. The samples were assayed at various concentrations, and inhibition percentage was calculated using the following formula: ((1 − (*A*_sample_ − *A*_sample blank_)/(*A*_control_ − *A*_control blank_)) × 100, where (*A*_sample_ − *A*_sample blank_) is the difference in the absorbance of a sample with or without 10 mM sodium nitroprusside, and (*A*_control_ − *A*_control blank_) is the difference in the absorbance of the PBS control with or without 10 mM sodium nitroprusside. Percentage inhibition was plotted against sample concentrations, and IC_50_ values denoted the concentration of sample required to scavenge 50% of NO free radical.

### 2.13. Acetyl Cholinesterase (AChE) Inhibition 

The enzymatic activity was measured using a slightly modified version of the method described by Aazza *et al.* [25]. Briefly, 50 µL of buffer 0.1 M (pH 8), 25 µL of extract dissolved in ethanol with different concentrations, and 25 µL of 0.22 U/mL of AChE enzyme (Sigma Aldrich, St. Louis, MO, USA) were mixed. After 15 min incubation at 37 °C, 25 µL of 15 mM acetylthiocholine iodide (AChI) (Sigma Aldrich, St. Louis, MO, USA) and 125 µL of 3 mM 5,5’-dithiobis [2-nitrobenzoic acid] (DTNB) (Sigma Aldrich, St. Louis, MO, USA) were added, and the resulting mixture was incubated for 30 min at room temperature. The absorbance of the mixture was measured at 405 nm using a microplate reader (Tecan Infinite M200, Tecan, Austria). The inhibitory effect of the test compound was determined by comparison to the negative control: Inhibition percentage (*I*%) = ((*A*_0_ − *A*_1_)/*A*_0_)) × 100, where A_0_ refers to the absorbance of the blank sample, and A_1_ to the absorbance of the sample. The test was repeated three times. The inhibition of the enzyme activity was expressed as IC_50_ (the concentration of the sample required to inhibit 50% of enzyme), which was calculated by a linear regression analysis.

### 2.14. Simulated Gastrointestinal Juice

The *in vitro* gastric system simulating the digestive process of the mouth and stomach was performed as previously described by Melo *et al.* [26]. All the components of the saliva, gastric and intestinal fluids were purchased from Sigma-Aldrich (Madrid, Spain) or WWR (Lisbon, Portugal). The assay started with exposure of the aqueous extracts (10 mL) to simulated saliva: KCl (89.6 g/L); KSCN (20 g/L); NaH_2_PO_4_ (88.8 g/L); Na_2_SO_4_ (57 g/L); NaCl (175.3 g/L); NaHCO_3_ (84.7 g/L); urea (25 g/L); 290 mg of α-amylase; 15 mg of uric acid; 25 mg mucin, pH 6.8 ± 0.2, for 5 min at 37 °C and 60 rpm. An aliquot was then taken for flavonoid quantification and antioxidant activity determination. After 5 min, an artificial gastric fluid: 1.884 mL NaCl (175.3 g/L); 0.36 mL NaH_2_PO_4_ (88.8 g/L); 1.104 mL KCl (89.6 g/L); 2.16 mL CaCl_2_·2H_2_O (22.2 g/L); 1.2 mL NH_4_Cl (30.6 g/L); 0.09 mL HCl 37% (*w/w*); 1.2 mL glucose (65 g/L); 1.2 mL glucuronic acid (2 g/L); 0.408 mL urea (25 g/L); 1.2 mL glucosamine hydrochloride (33 g/L); 0.12 g bovine serum albumin; 0.3 g pepsin; 30 mL mucin (12 g/L), pH 2.5, was added to the saliva medium, and incubation was continued for 2 h at 37 °C and slight agitation (60 rpm). An aliquot was then withdrawn again for flavonoid quantification and antioxidant activity determination. To simulate the gut environment, an artificial intestinal fluid (pH 6.5), consisting of 2.4 mL NaCl (175.3 g/L), 2.4 mL NaHCO_3_ (84.7 g/L), 0.6 mL KH_2_PO_4_ (8 g/L), 0.378 mL KCl (89.6 g/L), 0.6 mL MgCl_2_ (5 g/L), 0.0108 mL HCl 37% (*w/w*), 0.24 urea (25 g/L), 0.54 mL CaCl_2_·2H_2_O (22.2 g/L), 0.06 g BSA (bovine serum albumin), and 0.54 g pancreatin, and 0.09 g lipase was added to the previous gastric mixture. The bile fluid consisted of 1.2 mL NaCl (175.3 g/L), NaHCO_3_ (84.7 g/L), 0.168 mL KCl (89.6 g/L), 0.006 mL HCl 37% (*w/w*), 0.4 mL urea (25 g/L), 0.4 mL CaCl_2_·2H_2_O (22.2 g/L), 0.072 g BSA, and 1.2 g bile (0.6 g porcine bile, 0.6 g bovine bile). The sample extracts were exposed to the intestinal fluid for 2 h at 37 °C and 60 rpm. An aliquot was then taken for the quantification of flavonoids and determination of antioxidant activities. Three independent experiments were carried out.

### 2.15. Statistical Analysis

Statistical analysis was performed with the Statistical Package for the Social Sciences (SPSS) version 23.0 software (SPSS Inc., Chicago, IL, USA). Statistical comparisons were performed using the one-way analysis of variance followed by Tukey multiple comparisons. The level of significance was set at *p* < 0.05. Correlations between phenol and flavonoid content and antioxidant activity were determined by the Spearman correlation coefficient (r) at a significance level of 95% (*p* < 0.01). Paired Student’s *t*-test was used to determine differences between evaluated samples at significance level of 5% and 1%.

## 3. Results

### 3.1. Phenol and Flavonoid Content

The total phenolic (mg CAE/100 g, d.w.) concentrations recorded for the sample extracts from the various parts of *D. simplex* and *D. harra* species are presented in Table 1. The results revealed that phenolic concentrations depended on either the species or organ from which the extract was obtained. The highest rates of phenolic content were recorded for the seeds and flowers of *D. simplex* and *D. harra*, respectively (Table 1).

The highest amounts of flavones and flavonols were recorded in the seeds of *D. simplex* and *D. harra* (2422.4 and 1922.6 mg rutin equivalent (RE)/100 g, d.w., respectively), which were immediately followed by the flowers (2061.0 and 1461.1 mg RE/100 g, d.w., respectively) and siliques (755.5 and 909.1 mg RE/100 g, d.w., respectively) (Table 1). 

Flavanones and di-hydroflavonols were presented in all organs of *D. simplex* and *D. Harra*, although in much lower concentrations compared to flavones and flavonols (Table 1). The flowers and leaves of *D. harra* yielded into the highest amounts of flavanones and di-hydroflavonols (149.8 and 132.1 mg Naringenin Equivalent (NE)/100 g, d.w., respectively). In *D. simplex*, the stems presented the highest amounts of this group of compounds (119.8 mg NE/100 g, d.w.). Overall, and taking all the plant organs under investigation into account, *D. harra* showed significantly higher concentrations of flavanones and di-hydroflavonols than *D. simplex*, except for the stems (Table 1).

The amounts of phenols, flavones and flavonols recorded in *D. simplex* and *D. harra* were not significantly different (*p* < 0.05; Student’s *t*-test). The plant species showed, however, significantly different (*p* < 0.05, Student’s *t*-test) results in terms of di-hydroflavonol and flavanone contents, with higher concentrations observed for *D. harra*.

### 3.2. Antioxidant and Acetylcholinesterase Inhibitor Activities

The results revealed that the seeds and flowers of *D. simplex* presented the highest rates of free radical scavenging activities. The seeds presented the highest capacities to scavenge ABTS (IC_50_ = 0.35 mg/mL), superoxide anion radical (IC_50_ = 0.46 mg/mL) and NO radical (IC_50_ = 1.37 mg/mL) activities (Table 2). Furthermore, the seeds and flowers showed the highest capacities of scavenging DPPH free radicals (IC_50_ = 0.31 and 0.41 mg/mL, respectively). The flowers had the best capacity for scavenging hydroxyl free radicals (IC_50_ = 0.49 mg/mL), and the siliques presented the best total antioxidant activity measured from the capacity of reducing molybdenum anion (38.76 mg Ascorbic Acid Equivalent (AAE)/100 g). The flowers of *D. simplex* were, on the other hand, the best acetylcholinesterase inhibitors (IC_50_ = 0.42 mg/mL).

Concerning *D. harra*, the results revealed that while the seeds were the best scavengers of hydroxyl free radicals (IC_50_ = 0.48 mg/mL), the flowers were the best scavengers of ABTS, DPPH free radicals and superoxide anion free radicals (IC_50_ = 0.37 mg/mL; IC_50_ = 0.86 mg/mL and IC_50_ = 0.79 mg/mL, respectively) (Table 3). The findings also indicated that the leaves presented the highest rate of NO radical scavenging activity (IC_50_ = 1.18 mg/mL). The seeds and leaves were also noted to present the best chelating activities (IC_50_ = 1.58 and 1.80 mg/mL, respectively). The leaves showed the best total antioxidant activity measured through the capacity for reducing molybdenum anion (29.46 mg AAE/100 g). The leaves were also noted to be the best acetylcholinesterase inhibitors (IC_50_ = 1.18 mg/mL).

Total antioxidants were measured based on the reduction of molybdenum, and the results revealed that correlations only with total phenols, and not with flavonoids. In ABTS, free radical scavenging, antioxidants are involved in the redox mechanism of organisms against the free radical-mediated oxidation, which relies on the reduction of the blue-green cation radical ABTS^+•^ [27]. Although a redox mechanism is involved in the total antioxidant method with different reagents, different results were obtained (Table 4). In the ABTS method, there was an inverse correlation between the IC_50_ values and phenol and flavonoid amounts. The molybdenum reduction method, on the other hand, showed correlations only with phenol amounts.

Concerning *D. harra*, and for the superoxide anion radical scavenging, the inverse correlation was observed only between the IC_50_ value and di-hydroflavonol and flavanone amounts, but not with the other phenols as compared to *D. simplex* (Table 4). For nitric oxide scavenging, positive correlations were observed between IC_50_ values and flavonoids and phenol amounts. This was, however, not observed for *D. simplex*.

The flower extracts from *D. simplex* and *D. harra* presented the highest rates of acetylcholinesterase inhibition capacity (IC_50_ = 0.42 mg/mL and IC_50_ = 0.72 mg/mL, respectively) (Table 2). To the authors’ knowledge, the present study is the first to report on the *in vitro* inhibition of acetylcholinesterase by different organs of *Diplotaxis*. A negative correlation was observed between the IC_50_ values and phenol, flavone and flavonol amounts in *D. simplex*. For *D. harra*, such a correlation was observed only with di-hydroflavonol and flavanone amounts (Table 4). The results did not reveal significant differences between the two species, except in terms of nitric oxide scavenging ability (*p* < 0.05; t-Student’s *t*-test), with the precedence of *D. harra* (Table 2 and Table 3). 

### 3.3. Digestion

**Figure 1:**
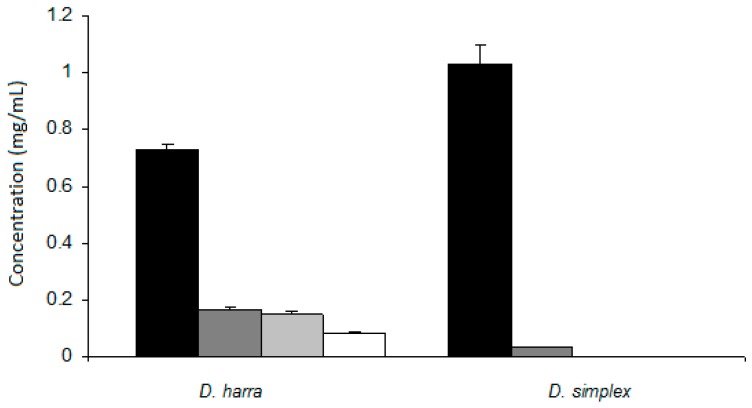
Concentration of flavonols and flavones before 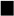 and after contact with artificial saliva 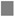, gastric 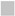 and intestinal fluid 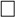.

**Figure 2:**
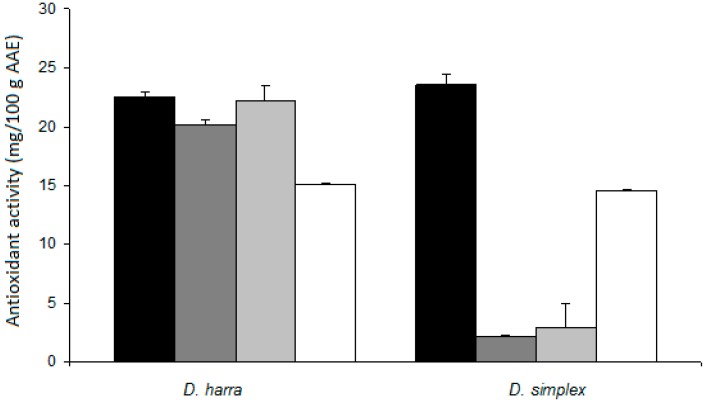
Antioxidant activity of extracts before 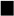 and after contact with artificial saliva 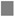, gastric 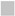 and intestinal fluid 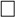.

In the present work, the *in vitro* gastric system simulating the digestive process of the mouth and stomach was developed to investigate compositional and behavioral changes of the flower aqueous extracts of *D. simplex* and *D. harra*. Flavonoid amount and total antioxidant was determined, and the results are presented in Figure 1 and Figure 2, respectively.

The amounts of flavones and flavonols presented in the aqueous extracts of *D. harra* underwent a drastic decrease after 5 min of contact with the simulated saliva, and then remained practically stable in the artificial gastric and intestinal fluids (Figure 1). The decrease of flavonoid content in the *D. simplex* extracts after contact with simulated saliva was much more drastic when compared to the samples of *D. harra* (Figure 2). Compared to their initial concentration before contact with the simulated saliva, only 15% of *D. harra* flavonoids were recovered in the simulated saliva, whereas 16% and 13% were recovered in the artificial gastric and intestinal media, respectively. These recovery rates were indicative for the considerable loss of flavonoids caused by exposure to saliva.

In *D. simplex*, the decrease of flavonoid concentration was even higher when compared to the values recorded for *D. harra* (Figure 1). After 5 min of contact with simulated saliva, the recovery of flavonoids was only 3%. After that, flavonoids were present only in trace amounts.

Two main factors contributed to the quantification of flavonoids and not total phenols: (a) great interferences were detected, hampering the quantification of total phenol by the Folin–Ciocalteau method; (b) derivatives of phenolic acids account for one third of the total intake of polyphenols in our diet, whereas flavonoids account for the remaining two thirds [28]. According to the results of the present study, more than 50% of phenols were flavonoids (Table 1).

The antioxidant activity was measured through the method of molybdate reduction. The antioxidant activity of *D. harra* decreased after the action of the simulated saliva but was much more pronounced after the action of intestinal fluid. The decrease of antioxidant activity was not as drastic as observed for the flavonoid content (Figure 1). Other components, which could not be determined in the present work, might be responsible for the antioxidant activity. In fact, the method used in this work revealed a correlation between the antioxidant activity and phenol or flavonoid content in *D. harra* (Table 4). However, in *D. simplex*, a significant decrease of antioxidant activity was observed after the contact of flavonoids with simulated saliva. In this case, and unlike *D. harra*, no correlation between antioxidant activity and flavonoids was observed (Table 4). Further studies are needed to investigate the increase of the antioxidant activity in intestinal fluid since flavonoids were practically absent. 

## 4. Discussion

### 4.1. Phenol and Flavonoid Content

Several studies have investigated the total phenols in *Diplotaxis* DC [12] using the same methodology employed in the present work, which is based on the reduction of molybdenum of Folin–Ciocalteau reagent. The literature indicates that flowers had higher concentrations of the total polyphenols than leaves. Nevertheless, the amounts recorded in this study were higher than those previously reported in the literature. Such differences may be related to the different standards used for the quantification of total polyphenols. In fact, while this work used caffeic acid, previous studies used gallic acid. The choice of caffeic acid as a standard was previously made in several researchers, including [12], and was guided by the fact that this standard was noted to have higher peak areas when compared to the remaining polyphenols previously separated and identified by high performance liquid chromatography (HPLC). The same authors did not, however, report on gallic acid as one of the polyphenols separated and identified by HPLC. However, harvesting place of plant material, edaphic and climatic conditions, and plant development stage might also be factors that contributed to the different results observed [12].

The flowers and leaves of *D. simplex* had higher amounts of polyphenols than those of *D. harra* (Table 1). The presence of relative high amounts of phenols in these organs may be useful for attracting pollinating agents as well as screening the amount of UV radiation reaching the earth [29]. Those results are in disagreement with the findings previously reported by Falleh *et al.* [12] who found that only the stems of *D. simplex* had higher concentrations of phenols. In our case, the concentration of phenols in this organ was practically the same. The phenols from seeds and roots were not quantified by Falleh *et al.* [12]. In our work, the seeds also showed relatively high amounts of phenols (Table 1). The seeds of both species had similar amounts of polyphenos (higher than 2600 mg CAE/100 g). Such concentrations were closer to those found in flowers (Table 1).

The seeds, flowers and leaves of *D. simplex* had higher amounts of flavones and flavonols than *D. harra* organs, which is in disagreement with the results reported by [12] for the same organs. The latter study did not study the seeds. The concentrations of flavonols and flavones found in the present work were also superior to those reported by some authors [12,30]. The differences between the results of the present study and those reported in those previous works could be attributed to the different standards used in quantification of polyphenols content, flavones and flavonols. While those authors [12,30] used catechin as a standard, the present work used rutin acid. Furthermore, the siliques, roots and stems of *D. harra* were noted to display higher concentrations of flavones and flavonols than *D. simplex*. Opposite results were observed for the seeds, flowers and leaves (Table 1).

Flavanones and di-hydroflavonols were not quantified by [12,30] for Tunisian plants. In fact, no previous data are currently available for use as possible baseline for comparison. The results revealed that most of the flavonoids found in *Diplotaxis* were flavonols, which is in line with previous reports [6,8] showing that flavonols and flavones were found at higher concentrations than flavanones and di-hydroflavonols. 

### 4.2. Antioxidant and Acetylcholinesterase Inhibitor Activities

Due to the lack of a validated and standardized method that can accurately determine the antioxidant capacity of foods, more than one analytical assay should be used. There are two main approaches to determining *in vitro* antioxidant activities of the samples: (a) the inhibition of lipid oxidation in different systems; and (b) the ability to scavenge free radicals species [25]. In the present work, we focused on methods based on the scavenging of free radicals, such as ABTS, DPPH, superoxide anion, hydroxyl and nitric oxide radicals. The capacity for scavenging some of these radicals, such as ABTS and DPPH, may be criticized because they are non-physiological free radicals. However, and due to their simplicity, these methods have almost always been used in practically all works involving the determination of antioxidant of samples and are, therefore, considered reference methods.

The hydroxyl radical is an extremely reactive species that reacts at a high rate with all surrounding molecules, such as proteins, lipids, nucleic acids, and sugars. Assays involving hydroxyl radical scavenging capacity estimation have been criticised because these radicals can recombine and quickly form hydrogen peroxide, with the steady-state concentration of hydroxyl radical being practically zero [31].

Superoxide anion radicals are produced as metabolism by-products and are responsible for the formation of other toxic reactive oxygen species, such as hydrogen peroxide, peroxynitrite, and hydroxyl radicals. Compounds with the capacity for scavenging superoxide anion radical will be of high interest because they will prevent the formation of those reactive oxygen species, which are deleterious for cells and tissues, avoiding, therefore, numerous chronic diseases, such as cancer, cardiovascular diseases, *diabetes mellitus*, gastric ulcer, and neurological disorders [32].

Nitric oxide has a number of physiological effects, including the control of blood pressure, neural signal transduction, platelet function, antimicrobial and antitumor activities. Infections and inflammations are often associated with high nitric oxide formations, which may induce several undesired and deleterious effects. Under aerobic conditions, the nitric oxide molecule reacts with the oxygen to produce intermediates, such as NO_2_, N_2_O_4_, N_3_O_4_, and stable nitrate and nitrite products, as well as peroxynitrite (reactive oxygen species) when reacting with superoxide [33]. Compounds with the capacity of scavenging nitric oxide can be considered as possessing antioxidant and anti-inflammatory properties.

Compounds with the capacity of chelating metal ions may also be considered as antioxidants since they can retard the rate of radical initiation reactions [27].

By chelating metal ions, phenols, such as flavonoids, generally prevent metal-catalyzed free radical generation. Flavonoids can interact with metal ions with the 3’,4’-dihydroxy group located on the B ring and the 3 or 5-hydroxy and 4-carbonyl group in the C ring [34]. The absence of correlation between flavones or flavonols and chelating activities may indicate that those hydroxyl groups are bonded to other molecules, such as carbohydrates. The activity found in those samples may only be attributed to other metabolites that are also metal chelating ions.

Acetylcholinesterase is the principal enzyme involved in the hydrolysis of acetylcholine. The great reduction of this neurotransmissor in the cerebral cortex is a significant factor in Alzheimer’s disease. Therefore, the use of compounds with the capacity of inhibiting that enzyme has been considered as a promising strategy for the treatment of neurological disorders, such as Alzheimer’s disease, senile dementia, ataxia and myasthenia gravis, in which a deficit in cholinergic neurotransmission is involved [25].

The capacity for scavenging ABTS free radicals by *D. simplex* and *D. harra* were already reported by Falleh *et al.* [12]. However, when they analyzed the antioxidant capacity measuring the prevention of β-carotene bleaching by *Diplotaxis* extracts, *D. simplex* generally showed a better chelating capacity, although depending on the fractions used. The same authors have also reported that the antioxidant activity was organ-dependent, with flower extracts being more active than stems or leaves. They attributed these results to the highest amounts of phenols detected in that organ.

Despite the antioxidant and anti-acetylcholinesterase activities of extracts, they were inferior to those observed for the positive controls assayed previously by Boulanouar *et al.* [35] and Albano *et al.* [36], respectively.

The results of the present study revealed negative correlations between IC_50_ values and total phenols and flavonols and flavanones, particularly for *D. simplex*, which were independent of the method used, except for chelating activity (Table 4). Such results are consistent with the hypothesis proposed by Falleh *et al.* [12] attributing those activities to the presence of phenols.

So far, and to the authors’ knowledge, this study is the first to report on the *in vitro* inhibition of acetylcholinesterase by different organs of *D. simplex* and *D. harra* and to correlate it with phenol contents. Phenols, including flavonoids, are known to possess acetylcholinesterase inhibitory as well as antioxidant activities. These two properties could open new promising opportunities for the development of multi-potent drugs for Alzheimer disease treatment [37].

In the absence of a correlation between phenols, flavones, flavonols, flavanones and di-hydroflavonol amounts and total antioxidant activities or a positive correlation between the amounts of the same compounds and the IC_50_ values of the NO scavenging activity, it seems that these properties found for *D. harra* should be attributed to other compounds and not only to phenols. In fact, Fernández-León *et al.* [38] have previously reported on a positive correlation between the antioxidant activity and glucosinolate content in two savoy cabbages. Glucosinolates are also present in *Diplotaxis* spp., which can contribute to the results obtained for *D. harra* under investigation [1].

### 4.3. Digestion

Reactive oxygen species are associated with a wide range of degenerative diseases, including cancer, cardio- and cerebrovascular diseases, immune system decline, brain dysfunction, and cataract formation. Epidemiological studies have demonstrated that the consumption of diets rich in vegetables and fruits is associated with lower incidence and mortality rates [39,40].

Some species of *Diplotaxis*, such as *D. harra*, are consumed as rocket salad. The antioxidant, anti-inflammatory, and acetylcholinesterase inhibitor activities of *D. simplex* and *D. harra* have been demonstrated in the literature, including the present work and several other previous reports [10,12,31]. These properties have been attributed to the phenolic compounds. Although these *in vitro* biological properties have been observed in diverse organs of *Diplotaxis* spp., further studies are needed to investigate whether they maintain their status or undergo alterations after ingestion.

In some artificial fluids, including that of the present work, flavonoids were present in small amounts that were so difficult to quantify. The flavonoids of the extracts in the presence of nitrogenous compounds, such as urea and ammonia, or biopolymers, such as mucin, which are present in the simulated saliva, may have undergone several chemical alterations, responsible for the results obtained [28]. A critical point of the present work was the time spent during the contact of the extract with the simulated saliva, because liquids spend only few seconds before being swallowed. In our case, the extract was in contact with the simulated saliva for 5 min. If alterations of the flavonoid content can occur in the simulated saliva, as was the case in our study (Figure 1), this would not be very important particularly if the assays take the short time of contact into consideration (few seconds and not minutes). Nevertheless and besides this procedure, the results may indicate that, for solid foods, in which the contact time between food and saliva is higher, flavonoids may undergo important chemical alterations. The results also demonstrated that, for *D. harra*, the artificial gastric and intestinal media did not exert a significant effect on those flavonoids that were not altered in the simulated saliva.

The decrease of the antioxidant activities was not as drastic as the one observed for flavonoid content, which suggested that other components, not determined in the present work, could be responsible for the antioxidant activities.

## 5. Conclusions

The results of the present study revealed that phenols and flavonoid content in *D. simplex* and *D. harra* extracts were dependent on the plant parts used, with the seeds and flowers being the richest organs in these compounds. More than 50% of total phenols were flavonoids, independently of the plant part used and in both species. Flavonols, flavones predominated over flavanones and di-hydroflavonols. There were not significant differences between the concentrations of phenols and flavonols and flavones of *D. harra* and *D. simplex*. Di-hydroflavonols and flavanones were significantly higher in *D. harra* than in *D. simplex*. The seeds and flowers generally presented the best antioxidant and acetylcholinesterase inhibition abilities. No significant differences were observed between antioxidant and acetylcholinesterase inhibition activities of *D. harra* and *D. simplex*. The sole significant difference was observed in their nitric oxide scavenging capacity, which was higher in the *D. harra* extracts.

A great loss of flavonoids was observed after the contact of the aqueous extracts of *D. harra* and *D. simplex* with the simulated saliva. In artificial stomach and intestinal fluids, the losses of those compounds were not as pronounced as the ones observed after exposure of *D. harra* extracts to simulated saliva. The deep decrease of flavonoids observed did not correspond to an accentuated reduction of activities, when measured through the method of molybdate, particularly in *D. harra* extracts, since for *D. simplex* extracts a significant decrease of activities was observed with a high increase in the artificial fluid. These results may indicate the presence of different compounds responsible for the tested activities, which require further investigations. 

## Figures and Tables

**Table 1:** Phenol content (mg caffeic acid equivalent/100 g, dry weight) found in plant extracts (mean ± standard error).

Organ	*D. simplex*	*D. harra*	*D. simplex*	*D. harra*	*D. simplex*	*D. harra*
	Phenols	Phenols	Flavonols	Flavonols	Di-hydroflavonols	Di-hydroflavonols
Seeds	2691.7 ± 70.1 ^b^	2694.5 ± 70.2 ^a^	2422.4 ± 60.5 ^a^	1922.6 ± 37.8 ^a^	24.1 ± 9.3 ^d^	75.9 ± 10.7 ^c^
Flowers	3433.4 ± 70.1 ^a^	2647.2 ± 70.2 ^a^	2061.0 ± 60.5 ^b^	1461.1 ± 37.8 ^b^	95.4 ± 9.3 ^a, b^	149.8 ± 10.7 ^a^
Leaves	1250.2 ± 70.1 ^c^	1017.0 ± 70.2 _c_	503.9 ± 60.5 ^d^	383.7 ± 37.8 ^e^	83.2 ± 9.3 ^b, c^	132.1 ± 10.7 ^a^
Siliques	1416.8 ± 70.1 ^c^	1371.2 ± 70.2 ^b^	755.5 ± 60.5 ^c^	909.1 ± 37.8 ^c^	63.6 ± 9.3 ^c^	122.6 ± 10.7 ^a, b^
Roots	446.8 ± 70.1 ^d^	684.6 ± 70.2 ^d^	348.5 ± 60.5 ^d^	448.0 ± 37.8 ^e^	27.2 ± 9.3 ^d^	70.8 ± 10.7 ^c^
Stems	499.1 ± 70.1 ^d^	502.4 ± 70.2 ^d^	344.8 ± 60.5 ^d^	592.2 ± 37.8 ^d^	119.8 ± 9.3 ^a^	94.7 ± 10.7 ^b, c^

In the column, different letters mean significant differences by the Tukey’s multiple range test (*p* < 0.05).

**Table 2:** Antioxidant activities of plant extracts of *D. simplex* expressed in IC_50_ (mg/mL) for ABTS, DPPH, superoxide, hydroxyl and NO scavenging activities, chelating activity and acetylcholinesterase inhibitor activity. Total antioxidant activity evaluated through the molybdate method is expressed as mg ascorbic acid equivalent/100 g, dry weight). (Mean ± standard error).

*Diplotaxis simplex*								
Organ	ABTS	DPPH	Superoxide	Hydroxyl	NO	Chelating	Molybdate	Acetylcholinesterase
Seeds	0.35 ± 0.02 ^e^	0.31 ± 0.49 ^c^	0.46 ± 0.18 ^d^	0.66 ± 0.05 ^c^	1.37 ± 0.97 ^d^	nd	23.53 ± 0.96 ^c^	2.33 ± 0.31 ^c^
Flowers	0.45 ± 0.02 ^d^	0.41 ± 0.49 ^c^	0.94 ± 0.18 ^c, d^	0.49 ± 0.05 ^d^	4.47 ± 0.97 ^c^	1.46 ± 0.38 ^c^	35.92 ± 0.96 ^b^	0.42 ± 0.31 ^d^
Leaves	0.69 ± 0.02 ^c^	3.67 ± 0.49 ^b^	1.00 ± 0.18 ^c^	0.66 ± 0.05 ^c^	6.31 ± 0.97 ^c^	0.41 ± 0.38 ^d^	20.50 ± 0.96 ^d^	3.04 ± 0.31 ^c^
Siliques	0.64 ± 0.02 ^c^	3.88 ± 0.49 ^b^	2.06 ± 0.18 ^b^	1.02 ± 0.05 ^b^	11.96 ± 0.97 ^b^	3.73 ± 0.38 ^b^	38.76 ± 0.96 ^a^	3.16 ± 0.31 ^c^
Roots	1.68 ± 0.02 ^a^	5.03 ± 0.49 ^b^	2.29 ± 0.18 ^a, b^	1.00 ± 0.05 ^b^	38.47 ± 0.97 ^a^	6.29 ± 0.38 ^a^	22.12 ± 0.96 ^c, d^	4.01 ± 0.31 ^b^
Stems	0.85 ± 0.02 ^b^	15.91 ± 0.49 ^a^	2.60 ± 0.18 ^a^	1.17 ± 0.05 ^a^	nd	2.46 ± 0.38 ^c^	12.34 ± 0.96 ^e^	5.44 ± 0.31 ^a^

nd: not determined. Values in the same column followed by the same letter are not significant different by the Tukey’s multiple range test (*p* < 0.05).

**Table 3:** Antioxidant activities of plant extracts of *D. harra* expressed in IC_50_ (mg/mL) for ABTS, DPPH, superoxide, hydroxyl and NO scavenging activities, chelating activity and acetylcholinesterase inhibitor activity. Total antioxidant activity evaluated through the molybdate method is expressed as mg ascorbic acid equivalent/100 g, dry weight). (Mean ± standard error).

*Diplotaxis harra*								
Organ	ABTS	DPPH	Superoxide	Hydroxyl	NO	Chelating	Molybdate	Acetylcholinesterase
Seeds	0.35 ± 0.03 ^e^	2.00 ± 0.16 ^d^	2.24 ± 0.20 ^b^	0.48 ± 0.05 ^d^	5.92 ± 0.34 ^a^	1.58 ± 0.13 ^c^	26.62 ± 0.58 ^b^	3.13 ± 0.21 ^b^
Flowers	0.37 ± 0.03 ^e^	0.86 ± 0.16 ^f^	0.79 ± 0.20 ^d^	0.96 ± 0.05 ^c^	4.60 ± 0.34 ^b^	3.05 ± 0.13 ^b^	22.50 ± 0.58 ^d^	0.76 ± 0.21 ^d^
Leaves	0.92 ± 0.03 ^c^	5.47 ± 0.16 ^c^	2.29 ± 0.20 ^b^	1.11 ± 0.05 ^b^	1.18 ± 0.34 ^d^	1.80 ± 0.13 ^c^	29.46 ± 0.58 ^a^	2.28 ± 0.21 ^c^
Siliques	0.72 ± 0.03 ^d^	1.36 ± 0.16 ^e^	1.61 ± 0.20 ^c^	0.94 ± 0.05 ^c^	3.13 ± 0.34 ^c^	3.81 ± 0.13 ^a^	24.24 ± 0.58 ^c^	4.36 ± 0.21 ^a^
Roots	1.59 ± 0.03 ^b^	13.06 ± 0.16 ^a^	4.13 ± 0.20 ^a^	1.30 ± 0.05 ^a^	nd	nd	22.28 ± 0.58 ^d^	nd
Stems	4.13 ± 0.03 ^a^	6.92 ± 0.16 ^b^	0.81 ± 0.20 ^d^	0.84 ± 0.05 ^c^	nd	nd	26.01 ± 0.58 ^b^	nd

nd: not determined. Values in the same column followed by the same letter are not significant different by the Tukey’s multiple range test (*p* < 0.05).

**Table 4:** Pearson correlation coefficients among compounds and antioxidant activities.

*Diplotaxis simplex*				*Diplotaxis harra*		
	Phenol	Flavonols	Di-hydroflavonols	Phenol	Flavonols	Di-hydroflavonols
Phenol	1	0.922 **	-	1	0.934 **	-
Flavonols	0.922 **	1	-	0.934 **	1	-
Di-hydroflavonols	-	-	1	-	-	1
ABTS	−0.734 **	−0.680 **	-	−0.724 **	−0.521 *	-
DPPH	−0.715 **	−0.622 **	0.555 *	−0.745 **	−0.685 **	−0.556 *
Superoxide	−0.784 **	−0.780 **	-	-	-	−0.554 *
Hydroxyl	−0.828 **	−0.716 **	-	−0.586 *	−0.777 **	-
Nitric oxide	−0.751 **	−0.661 **	-	0.919 **	0.964 **	-
Chelating	-	-	−0.707 **	-	-	-
Total antioxidant	0.570 *	-	-	-	-	-
Acetylcholinesterase	−0.908 **	−0.761 **	-	-	-	−0.713 **

-: Not significant. * Correlation is significant at the *p* < 0.05 level. ** Correlation is significant at the *p* < 0.01 level.
